# Mobile Application to Promote Adherence to Oral Chemotherapy and Symptom Management: A Protocol for Design and Development

**DOI:** 10.2196/resprot.6198

**Published:** 2017-04-20

**Authors:** Joel Nathan Fishbein, Lauren Ellen Nisotel, James John MacDonald, Nicole Amoyal Pensak, Jamie Michele Jacobs, Clare Flanagan, Kamal Jethwani, Joseph Andrew Greer

**Affiliations:** ^1^ Department of Psychology and Neuroscience University of Colorado Boulder Boulder, CO United States; ^2^ Department of Epidemiology Harvard TH Chan School of Public Health Boston, MA United States; ^3^ Department of Psychology University of California, Los Angeles Los Angeles, CA United States; ^4^ Anschutz Medical Campus Department of Medicine University of Colorado Denver Denver, CO United States; ^5^ Center for Psychiatric Oncology and Behavioral Sciences Massachusetts General Hospital and Harvard Medical School Boston, MA United States; ^6^ Department of Psychiatry Massachusetts General Hospital and Harvard Medical School Boston, MA United States; ^7^ Partners Connected Health Partners HealthCare Boston, MA United States; ^8^ Department of Global Health and Population Harvard TH Chan School of Public Health Boston, MA United States; ^9^ Department of Dermatology Massachusetts General Hospital and Harvard Medical School Boston, MA United States

**Keywords:** telemedicine, neoplasms, mobile apps, medication adherence, self-administration, antineoplastic agents, ambulatory monitoring, mHealth, software design

## Abstract

**Background:**

Oral chemotherapy is increasingly used in place of traditional intravenous chemotherapy to treat patients with cancer. While oral chemotherapy includes benefits such as ease of administration, convenience, and minimization of invasive infusions, patients receive less oversight, support, and symptom monitoring from clinicians. Additionally, adherence is a well-documented challenge for patients with cancer prescribed oral chemotherapy regimens. With the ever-growing presence of smartphones and potential for efficacious behavioral intervention technology, we created a mobile health intervention for medication and symptom management.

**Objective:**

The objective of this study was to develop and evaluate the usability and acceptability of a smartphone app to support adherence to oral chemotherapy and symptom management in patients with cancer.

**Methods:**

We used a 5-step development model to create a comprehensive mobile app with theoretically informed content. The research and technical development team worked together to develop and iteratively test the app. In addition to the research team, key stakeholders including patients and family members, oncology clinicians, health care representatives, and practice administrators contributed to the content refinement of the intervention. Patient and family members also participated in alpha and beta testing of the final prototype to assess usability and acceptability before we began the randomized controlled trial.

**Results:**

We incorporated app components based on the stakeholder feedback we received in focus groups and alpha and beta testing. App components included medication reminders, self-reporting of medication adherence and symptoms, an education library including nutritional information, Fitbit integration, social networking resources, and individually tailored symptom management feedback. We are conducting a randomized controlled trial to determine the effectiveness of the app in improving adherence to oral chemotherapy, quality of life, and burden of symptoms and side effects. At every stage in this trial, we are engaging stakeholders to solicit feedback on our progress and next steps.

**Conclusions:**

To our knowledge, we are the first to describe the development of an app designed for people taking oral chemotherapy. The app addresses many concerns with oral chemotherapy, such as medication adherence and symptom management. Soliciting feedback from stakeholders with broad perspectives and expertise ensured that the app was acceptable and potentially beneficial for patients, caregivers, and clinicians. In our development process, we instantiated 7 of the 8 best practices proposed in a recent review of mobile health app development. Our process demonstrated the importance of effective communication between research groups and technical teams, as well as meticulous planning of technical specifications before development begins. Future efforts should consider incorporating other proven strategies in software, such as gamification, to bolster the impact of mobile health apps. Forthcoming results from our randomized controlled trial will provide key data on the effectiveness of this app in improving medication adherence and symptom management.

**Trial Registration:**

ClinicalTrials.gov NCT02157519; https://clinicaltrials.gov/ct2/show/NCT02157519 (Archived by WebCite at http://www.webcitation.org/6prj3xfKA)

## Introduction

### Background

Oral chemotherapies are increasingly prescribed as an alternative therapeutic delivery method to traditional intravenous chemotherapy. In 2008, an estimated 25% of the more than 400 chemotherapeutics being developed involved oral agents [1]. Oral chemotherapy provides many benefits, including ease of administration, convenience, and minimization of invasive infusions. In addition, research has shown that patients receiving cancer treatment prefer oral chemotherapy versus intravenous, given equal efficacy and toxicity [2,3]. Thus, oral chemotherapy medications offer flexible, home-based treatment that has been associated with improvements in quality of life by reducing interference with daily activities, heightening perceived control over treatment, and increasing convenience [2,4,5].

Despite this revolutionary shift in cancer care delivery, home administration presents unique challenges to patients and clinicians. Home administration results in less oversight, support, and symptom monitoring from clinicians as compared to directly observed infusion chemotherapy [6]. In turn, patients may waver with regard to their medication adherence, and treatment might not be as effective as intended. It is well documented that patients with cancer have suboptimal adherence to oral chemotherapy [7-9]. Researchers have observed wide variations in adherence to oral chemotherapy depending on method of assessment, reporting rates as low as 16% [8]. The importance of oral chemotherapy adherence cannot be overstated, as patients who have poor adherence are more likely to experience worse clinical outcomes, such as morbidity, recurrence, and mortality *.* Suboptimal adherence to oral chemotherapy results in poor quality care, presenting a significant public health concern. As oral chemotherapy plays a more prominent role in cancer treatment, it is important to address medication adherence and improve symptom management for patients [10].

Limited research has been conducted to examine intervention methods to improve adherence to oral chemotherapy. The few intervention studies conducted to date have proven inconclusive in their effectiveness in improving adherence and display methodological limitations such as nonrandomized design and small sample sizes [11]. Thus, further research is greatly needed to develop and test novel, theoretically grounded, accessible interventions to promote patient medication adherence behaviors and symptom management.

Intervention accessibility is enhanced with the use of mobile health (mHealth) technology for the delivery of health care and wellness support [12]. Specifically, mobile phones allow behavioral science researchers to implement ecological momentary interventions and ecological momentary assessments (EMIs, EMAs) to deliver interventions and gather data in real time, under convenient and accessible real world situations [13]. Smartphones present an opportunity to implement more sophisticated EMIs and gather more detailed EMAs than prior research that has used short message service technology through pagers or non-Internet enabled cellular devices [14,15]. Researchers are thus able to optimize the delivery of behavioral interventions and collect ongoing data with minimal burden to the patient and provider. A recent review indicates that leveraging mobile technologies to deliver accessible interventions can improve health behaviors in patients with cancer [16]. Additionally, there is a proliferation of efforts in the literature to develop smartphone mobile app-based medication adherence interventions across varying chronic illnesses [17-22]. Smartphone mobile apps provide an ideal platform to provide relevant patient support in a context of medication adherence. Thus, we developed a mobile app intervention, *C* hem *o* the *r* apy *A* ssistant (CORA), to improve medication adherence and symptom management for patients with cancer taking oral chemotherapies.

The aim of this paper is to describe the theoretically based development of a smartphone mobile app intervention for oral chemotherapy adherence and symptom self-management in a diverse cancer population. Experts in behavioral intervention technology and mHealth implementation science have called for contributions to the literature that detail the development process of mHealth interventions [23]. Approximately 30,000 to 90,000 health care–related apps are currently available to consumers, while the US Food and Drug Administration has reviewed approximately 100 [12]. In an effort to increase transparency in mHealth intervention development, we describe the theoretical framework that supports the use of modern mobile technology to address oral chemotherapy adherence and symptom management and we detail the iterative process that our research and development teams undertook to design and create the CORA mobile app. Specifically, we used a framework for mHealth intervention development detailed by Whittaker et al [24] to guide the creation of our mobile app intervention. Steps identified as leading to successful intervention development include theoretical conceptualization, formative research, pretesting, piloting, randomized controlled trial, and qualitative follow-up. The remainder of this paper details our conceptual model, design stages, and stakeholder engagement in qualitative formative research, as well as development, refinement, finalization, and piloting of our smartphone app for improving symptom management and adherence to oral chemotherapy. We conclude with a discussion of lessons learned throughout the process.

## Methods

### Setting

This mixed-methods, randomized, parallel assignment, intervention trial [ClinicalTrials.gov: NCT02157519] began in 2014 and is currently being conducted at the Massachusetts General Hospital (MGH) Cancer Center, an academic hospital comprised of 24 multidisciplinary disease centers. The study was approved by the Dana Farber/Harvard Cancer Center Institutional Review Board (IRB) and is supported by the Patient-Centered Outcomes Research Institute (IHS-1306-03616).

### Procedure

Phase I (the study being described here) consisted of the mobile app development process and initial results of acceptability that were completed in 5 steps. Phase II, currently underway, consists of a randomized controlled trial (RCT) to test acceptability, feasibility, and efficacy of the mobile app intervention in improving adherence to oral chemotherapy and symptom management in patients with cancer.

The 5-step development process in phase I consisted of identifying desired features, content, and functionality of a mobile app in an iterative process. Step 1 included using expert collaboration and theoretical framework to guide initial content development. In step 2, we conducted focus groups with key stakeholder groups to inform the study design and development of the mobile app. Step 3 involved the creation of wireframes for the initial app components (eg, adherence, symptom management, communication with treatment team, educational resources) and individual interviews with patients and clinicians to review the proposed app content using these screen blueprints. Step 4 consisted of designing, programming, and further refining the prototype of the app for testing. Last, step 5 entailed the finalization of the prototype for the RCT.

### Research, Development, and Stakeholder Teams

The multidisciplinary research team consisted of the principal investigator, co-investigators (expert clinicians and scientists specializing in psychology, oncology, and psychiatry), a project director, and 3 research assistants. The technical development team was comprised of programmers and project managers at Partners HealthCare Connected Health. Stakeholders served as expert consultants, including oncology clinicians, health care representatives, practice administrators, patients, and family members. All stakeholder groups reviewed the proposed study design and app features, providing initial feedback on acceptability of content and implementation of the intervention. Patients and family caregiver stakeholders also participated in alpha and beta testing, including qualitative interviews to assess the acceptability and ease of using the intervention.

### Step 1: Implementing a Theoretical Framework

As oncology clinicians are increasingly prescribing oral chemotherapy in cancer care settings, new concerns are emerging with respect to patients self-administering medications with high toxicity profiles. Patients and their family caregivers now bear the primary responsibility of administering care, often with less frequent follow-up and support from their health care team to ensure proper adherence and to monitor and manage symptoms. To address these salient issues and guide the development of CORA, our research and development team consulted the conceptual model by Murray et al [25] which identifies key elements of medication adherence. Factors contributing to medication adherence in chronic illness are best understood across patient, provider, and health care system levels. The model describes the interaction among these patient, provider, and health care system factors and their influence on the relationship between increased medication adherence and improved health outcomes. Four factors were identified as the most important barriers to oral chemotherapy adherence: complexity of medication regimens, symptom burden, poor self-management of side effects, and low clinician support. A description of how the present mobile app intervention was designed to intervene on these barriers to medication adherence and symptom management is provided in Table 1 and is the basis for the development of app content. By increasing patient engagement with treatment, providing simple medication reminders, assisting patients with symptom monitoring and management, and facilitating communication with the care team in the form of a low-burden, easily accessible, and user-friendly mobile smartphone app, barriers to optimal adherence to medication may be overcome.

**Table 1:** Assessing and intervening on the barriers to optimal medication adherence.

Barrier	How addressed in Chemotherapy Assistant (CORA)
Complex medication regimens	Personalized medication treatment plan: medication, dosage, frequency of drug, breaks/chemo holiday, medication reminder alerts.
Symptom burden	Symptom reporting: patients can report symptoms as they are occurring, receive tailored feedback, and send the symptom report to their care team.
Poor self-management of side effects	Symptom feedback: CORA asks questions to assess the severity and frequency of symptoms and provides feedback for managing symptoms and resources for contacting care team.
Low clinician support	Weekly symptom reports: symptom reports are sent to each patient’s care team on a weekly basis to inform clinical decision making.

### Step 2: Conducting Initial Focus Group Interviews With Key Stakeholders

#### Stakeholder Selection

To inform the study and development of the intervention, we sought out stakeholders both within and outside the local community, obtaining comprehensive feedback across levels of expertise in cancer. Drawing from a model of population-based patient-centered care, 32 stakeholders served as study collaborators and comprised 4 key stakeholder groups: (1) patient/families (n=8); (2) oncology clinicians (n=8); (3) cancer practice administrators (n=8); and (4) representatives of the health system, community, and society (n=8). We pulled from a diverse group of professionals, including pharmacists, health care leaders, lawyers, and patient advocates to inform decisions related to pharmacology, liability, health communication, and support resources. The patient and family member stakeholder group took place in person onsite at the MGH Cancer Center. For the other stakeholder groups, the interviews took place via teleconference call, as many of the participants were from organizations and care settings across the United States. Stakeholders were included as consultants in the study and were not consented as participants; therefore, we did not collect demographic or other personal information from the stakeholders.

The goal of these initial focus group interviews was to obtain feedback about the proposed study topic, design, and intervention. The stakeholder interview guide included the following topics: (1) perceived importance of monitoring of adherence to oral chemotherapy; (2) barriers to communication between patients and oncology team regarding management of side effects and medication adherence; (3) potential role of the mobile app to address barriers to quality of cancer care; (4) potential feasibility, acceptability, and usability of an mHealth intervention; (5) feedback on the overall study design; and (6) systems barriers and facilitators to implementation. Stakeholder conversations were heavily focused on logistics of implementation in the clinic.

#### Patient and Family Member Stakeholder Group

A total of 8 patient and family caregiver stakeholders participated in a focus group to provide feedback about their experiences relevant to the patient taking oral chemotherapy in order to promote user acceptability. Although the app is intended for use only by patients, we believed that family caregivers could provide additional insights as they are often highly involved in patient medication and symptom management. Key points from these focus groups included developing features that would optimize potential benefit and minimize burden. Both patients and family members strongly recommended a symptom monitoring feature with easily interpretable graphics to display symptom severity over time. In addition, patients and family members emphasized the importance of clear instructions within the app regarding how to address and follow-up on urgent versus nonurgent symptoms. This feedback was integral toward informing the development of the app and establishing a protocol to alert patients when they should immediately contact their care team after reporting a concerning symptom.

#### Oncology Clinician, Practice Administrator, and Health Care Representative Stakeholders

We conducted 3 separate focus group interviews via teleconference call for the oncology clinician, practice administrator, and health care representative stakeholders. These focus groups identified themes related to intervention content, randomized trial study design, and methods for implementation. Health care representatives suggested providing patients with relevant clinical resources in the app such as disease-specific social networking sites, as well as the importance of promoting patient-physician communication through symptom reporting. Practice administrators provided suggestions for capturing important outcomes and data management, from stratifying randomization in the RCT by line of chemotherapy to tracking participant usage of CORA. Oncology clinicians provided feedback on involving the care team without burden, as well as promoting participant engagement through incentivizing features such as a Fitbit device with activity tracking enabled in the mobile CORA app.

### Step 3: Creating Wireframes and Collecting Specific Feedback on App Components

#### Creation of Wireframes and Interview Guide

**Figure 1:**
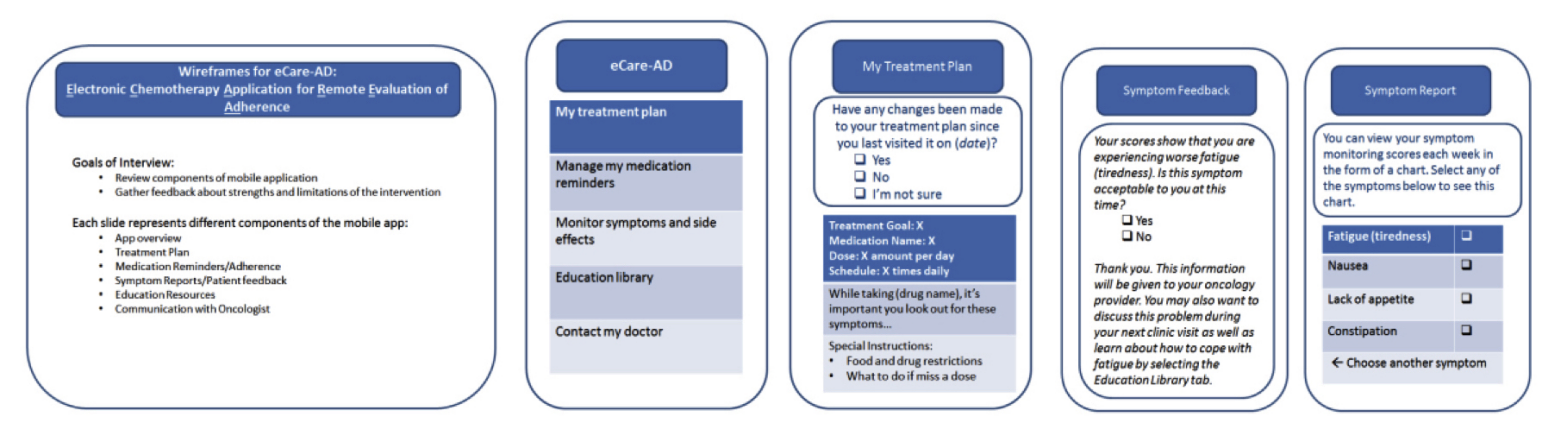
Wireframes for Chemotherapy Assistant mobile app.

In addition to conducting initial stakeholder focus groups, the research and design teams created content wireframes (ie, screen blueprints) to provide a visual guide for the components of CORA (see Figure 1). We created wireframes for the following mobile app sections: personalized treatment plan, medication reminder system, symptom reporting, education library, and notes and questions. The research team then used these wireframes to conduct individual qualitative interviews with 10 patients and 8 oncology clinicians at the MGH Cancer Center.

The semistructured interview guides for patients and clinicians addressed 3 domains: (1) components of the mobile app, (2) feasibility and usability of the mobile app, and (3) weekly in-app symptom assessments to be shared with the prescribing oncology clinician. Patients were asked to consider which features they would be likely to use in a mobile app to support medication taking. Clinicians were asked to provide input on the timing, frequency, and feasibility of receiving symptom assessment reports weekly, as well as potential barriers or burdens of this feature. All patient and clinician participants signed IRB-approved consent forms, and the interviews were audiorecorded.

#### Collecting Feedback From Massachusetts General Hospital Patient Participants

A total of 10 patients at the MGH Cancer Center participated in qualitative interviews to evaluate the acceptability as well as potential feasibility and usability of a mobile app intervention to help improve adherence to oral chemotherapy and symptom management. The majority of the patients were female (6 female, 4 male), white (8 white, 1 Asian, 1 Hispanic/Latino), married (7 married, 3 single), and well-educated (6 completed college or higher, 2 completed high school or GED) with a mean age of 58.40 (SD 8.02) years. All the patients had been prescribed oral chemotherapy for the treatment of either non–small cell lung cancer (n=4), breast cancer (n=2), prostate cancer (n=1), chronic myeloid leukemia (n=1), or multiple myeloma (n=1).

Goals of the individual interviews were to gauge patient interest in a mobile app intervention and gather feedback about suggested topics to include within the education section, aesthetics, and frequency of push notifications to patients. The patient participants also provided feedback on current problems and difficulties as well as their preferences for using CORA.

#### Collecting Feedback From Massachusetts General Hospital Oncology Clinicians

A total of 8 oncology clinicians (6 female, 2 male), including 4 physicians and 4 nurse practitioners who specialize in breast oncology (n=2), genitourinary oncology (n=3), thoracic oncology (n=2), and melanoma (n=1) at the MGH Cancer Center, also viewed the wireframes and completed qualitative interviews to inform the implementation of the mobile app intervention. Themes from these qualitative interviews included the structure and frequency of weekly symptom reports sent from the mobile app server to the clinician, incorporation of the intervention into a patient’s treatment plan, and mitigating risk involved with symptom reporting via CORA. These interviews were integral in establishing the practical methodology for successfully implementing this project locally, at the main site, before disseminating more widely at community affiliates.

### Step 4: Developing, Programming, and Refining the App

#### Technical Specifications and Process

The CORA mobile app was originally developed on the Titanium 3.5 (Appcelerator Inc) platform to ensure cross-platform functionality on both Apple iOS and Android devices. Later updates were developed on Titanium 5.0. While CORA was written primarily in JavaScript language, some custom features (using native functionality) were written in Objective-C and Java, such as the local notifications.

CORA was developed to minimize app-driven data and battery use. For example, symptom data and survey responses were programmed to be transmitted to the server once weekly. Analytics data were programmed to be sent once daily. In both cases, in the event of no Internet connection, CORA transmits the data at the next connection opportunity. Medication reminders were programmed as local notifications to ensure delivery independent of Internet connection. All app-related data are encrypted both on the smartphone and during data transmission, independent of the user phone’s level of encryption.

CORA is supported by a PHP/MySQL database, which is hosted on a LAMP (Linux, Apache, MySQL, Perl/PHP/Python) server that meets all Health Insurance Portability and Accountability Act (HIPAA) Security Rule requirements. The database collects and stores all user-inputted information and engagement metrics, tracking which pages each user visits and how long the user remains on a given page. User-inputted information is used to generate weekly symptom reports to providers and measure study outcomes.

CORA underwent code reviews and quality assurance testing with each code release. The entire study team, including developers, research staff, and clinicians, participated in the quality assurance process.

#### Alpha Testing

The development of CORA was conducted through an iterative process between the research and technical development teams. In addition to the focus group and qualitative interviews that were conducted before development, we gathered feedback from the patient and family stakeholders throughout the development process and following the release of the beta app.

Toward the conclusion of the development cycle, we invited members of the initial patient and family stakeholder group to participate in user acceptance testing (UAT). Participants with varying levels of comfort using technology were included in order to maximize generalizability. During UAT sessions, research and development staff observed users during their initial interactions with CORA. Participants were then asked to complete specific tasks within the demo app. For example, participants were asked, “How do you think you would go about adding your oral chemotherapy medication to this app?” Research and development staff then observed participants completing the different tasks. Participants were invited to share general feedback on CORA toward the end of the testing session, specifically on how intuitive they considered the tasks to be. We recorded participant responses, and this exercise helped the development team determine whether users would be able to navigate CORA intuitively on their own. Feedback from the UAT was then incorporated into the development cycle to improve the user experience within CORA. Prior to the finalization of the prototype, the research team (N=4) reviewed multiple iterations of CORA and provided aesthetic and functional feedback to the technical team on a weekly basis.

### Step 5: Finalizing the App Prototype

#### Beta Testing

To obtain and incorporate more long-term user feedback, the research team considered the first 5 participants enrolled in the subsequent RCT phase who received the CORA app intervention as beta testers. After the first 5 participants completed the 12-week follow-up assessment, research staff conducted qualitative interviews with them to learn about their use of the CORA app. The 5 participants completed a 20-minute, semistructured interview about their experiences using CORA. Interview questions evaluated the feasibility, usability, and aesthetics, allowing users to suggest changes or enhancements.

Additionally, the research team conducted qualitative interviews with 5 oncology clinicians who had patients assigned to the intervention group. Upon completion of study procedures, these clinicians were interviewed about their conversations with patients pertaining to CORA, as well as interpretation and helpfulness of the weekly symptom reporting feature.

## Results

Feedback from stakeholders and beta testers highlighted specific features that they believed needed to be addressed in the app. Patients, caregivers, health care representatives, and practice administrators provided suggestions related to the patient experience of the app; oncology clinicians also made suggestions that would best enable oncology care teams to make use of the data that patients enter into the app. Key feedback provided by each group and the implementation of that feedback in the app is shown in Table 2.

**Table 2:** Stakeholder and beta tester feedback and implementation of that feedback.

Group	Quotes from stakeholder feedback	Implementation
Patients and families	“Connect patients with the same disease type for social support.”	Feature: Education Library Module—Resources and Social Networking. CORA includes a list of reputable, disease-specific resources for patients looking to connect with others.
Health care representatives	“Provide patients with anchors and definitions of symptoms so they can appropriately determine the severity and urgency of their symptoms.”	Feature: Symptom Reporting Module. When a patient reports a symptom, CORA asks several questions about the frequency and duration before providing tailored feedback.
Oncology clinicians	“The weekly symptom reports that are sent to clinicians should be concise and easy to understand.”	Feature: Symptom Reporting Trends Module. Weekly symptom reports provide a list of symptoms reported by the patient, as well as a color and numeric value (1-10) denoting severity.
Practice administrators	“Provide resources and contact information for patients to use when they miss a dose of their medication.”	Feature: Symptom Reporting Module—“touch to call clinical team” feature. Patients are provided with the study team contact information at baseline. Embedded in the symptom reporting feature is a “touch to call” button for their specific clinic.

The version of CORA delivered at the end of the development process described here is organized into 6 functional modules (Table 3). Each module is accessed via a navigation bar at the bottom of the app’s screen. In the Homepage module, a personalized medication treatment plan shows users their oral chemotherapy medication name and dosage and provides reminder alerts to take their oral chemotherapy at their self-selected daily dosage time. The Homepage also displays healthy recipe recommendations. The Symptom Reporting Module enables users to record concerning symptoms (eg, high fever) or more common symptoms (eg, fatigue) on a daily basis. Users can report severity of these symptoms on a continuous sliding scale from mild to severe. When users report symptoms, the module probes them to provide specific information about each symptom with multiple choice questions, and based on user responses, provides personalized management recommendations for that symptom. Additionally, if participants report concerning symptoms in the app such as high fever, the app recommends that the participant immediately call their doctor and provides the oncology on-call clinic number onscreen. Weekly summaries of user reported symptoms and severity, converted to a 0 to 10 value based on where the participant placed the slider on the scale, are compiled into automated report documents. The study team then sends these reports via secure email to users’ medical oncologists and oncology nurse practitioners. Trends in these symptoms are displayed on the CORA app in graphical format in the Symptom Reporting Trends module, showing users the severity of each symptom on each day it was reported. The Education Library module provides information about common symptoms and symptom management, nutrition, and links to online resources relevant for individuals with cancer, such as the Leukemia and Lymphoma Society’s online community discussion forum. To help users remember what they want to discuss at their next clinic visit, the Notes and Questions module provides a free-response text field to record questions for their oncologist. The Wearable Fitness Tracking Device module syncs data from Fitbit devices and displays daily step counts. Finally, the app also enables users to set daily step goals and displays progress toward their goals.

**Table 3:** Organization of Chemotherapy Assistant (CORA) modules and their components.

Module	Components
Homepage	Medication treatment plan, suggested healthy recipes.
Symptom reporting	Weekly and real-time symptom reporting for common symptoms and treatment side effects, with algorithms that personalize symptom management suggestions or enable participants to call their care team directly from the app.
Symptom reporting trends	Graphical display of weekly symptom trends, customized for each patient and their medical oncologist.
Education library	Symptom management, social networking and support resources, nutrition, and clinic contact information.
Notes and questions	Store notes and questions for future clinic visits.
Wearable fitness tracking device	Stream data from Fitbit devices to CORA, display daily step counts, and allow users to set daily step goals and display progress towards those step goals.

CORA is compatible with iOS [iPhone] and Android smartphone operating systems and all Fitbit wearable fitness tracker devices. Due to limited engineering resources, we could not support compatibility with other operating systems and wearable device brands in this version of CORA.

As we are now testing CORA in an RCT (since the trial is in progress, results of the RCT are not reported here), we continue to refine the app. For example, as newer iOS, Android, and Fitbit software is released, we make changes to the app to ensure continuing compatibility. We likewise address any software bugs that emerge by communicating with the programming team to determine the cause of and the solution to the bug. Once the new CORA version is ready, the research team guides participants through upgrading to that version and provides that version to new participants. We have created 7 updated versions of the app during the RCT phase (since August 2014): 4 updates addressed integration with third-party smartphone operating systems and 3 updates addressed bugs or minor improvements (eg, adding additional question logic to the medication treatment plan in the Homepage module) deemed necessary by the study team.

We also continue to engage with our stakeholders throughout the RCT phase with quarterly updates on progress of the study. Twice, we have asked stakeholders to provide feedback on aspects of the RCT, including whether any additional incentives should be provided to study participants and how best to engage clinicians in the study. After the RCT is complete, we will solicit final feedback from our stakeholders about the preliminary analyses and interpretation of the results, methods for improving the app for future uses, and plans for broader dissemination across care settings.

## Discussion

### Principal Findings

We developed a novel smartphone app to improve oral chemotherapy adherence and self-management of symptoms for patients with cancer. The app development was informed by a conceptual model that attributed poor oral chemotherapy adherence to patient, clinician, and health care system factors. Based on this model, we designed a prototype version of the app. In focus groups and individual qualitative interviews, we solicited feedback from stakeholder groups, including patients, patients family members, oncology clinicians, oncology practice administrators, and representatives of health care organizations. We also conducted user testing on an early version of the app to assess acceptability and usability. The feedback elicited from our stakeholders and testers informed ongoing refinement of the prototype. Having completed initial development, we are iteratively updating and improving the app based on feedback from stakeholders and participants in an ongoing RCT.

CORA addresses many challenges that patients and providers face with oral chemotherapy. Although oral chemotherapies are increasingly being developed and used in standard care [1,26] and patients prefer these treatments to intravenous chemotherapy [2,27], treatment with oral chemotherapy poses barriers for adherence and monitoring of side effects [28]. We aimed to improve adherence with CORA by providing automated medication reminders, personalized dosing and treatment information, and weekly questionnaires assessing adherence and symptoms. Patient responses on the weekly adherence questionnaires are sent to their care teams, which may help clinicians intervene when patients are struggling to take their medication as prescribed. Likewise, we aimed to address management of side effects with app features for reporting and tracking symptoms and side effects in real time. We included an ad hoc symptom questionnaire in the app for patients to log symptoms they are experiencing at any time. Simple graphs show users the trends in symptoms they have reported over time. As with the adherence questionnaires, we send symptom summaries to their respective clinicians so they can follow up with patients about new or chronic symptoms at their next scheduled visit or intervene sooner based on their clinical judgment.

We engaged stakeholders representing all types of individuals and organizations that could be influenced by the dissemination of our app. One stakeholder group was patients diagnosed with cancer, the intended future users of the technology. We also included family members as stakeholders, given that caregivers often administer medications and communicate with the care team. As our app aims to enhance how clinicians manage their patients taking oral chemotherapy, we included stakeholder groups of oncology clinicians and practice administrators. Finally, we included stakeholders representing health care institutions, including insurers, patient advocacy groups, research hospitals, and pharmaceutical companies. These organizations may influence future development, use, and dissemination of our app.

Feedback from experts in diverse fields ensured that our app is relevant for individuals and organizations involved in cancer care. For example, the clinician stakeholder group provided useful feedback about how to avoid overburdening clinicians with symptom reports from their patients. Their suggestions ultimately shaped the system that was implemented. We engaged stakeholders at every stage of development. Focus groups conducted before any development took place informed the modules of the app that were ultimately included. Reviewing wireframes and early app versions with patients and clinicians helped us address concerns with the look, feel, and use of the app before it was finalized. We are continuing to solicit feedback from stakeholder groups during the RCT phase to ensure that our app remains current with continuously changing trends in technology and health care. Additional focus groups conducted at the end of the RCT phase with each of the stakeholder groups will help us to plan our next steps for dissemination.

### Limitations

Our development process was not without limitations. First, the patient population at the academic hospital where we developed the app is homogeneous. Most patients identify as white and non-Hispanic and have higher income and education levels than average for the greater Boston area where the hospital is located. Furthermore, including only patients with iPhone or Android smartphones biases our sample toward younger, wealthier, and better educated individuals. Therefore, our findings in the development process and the RCT may not be applicable to broader populations. However, we did recruit a diverse sample of stakeholders, representing many disciplines and professions, community and academic medical centers, and geographical locations. Additionally, acknowledging this limitation we later deployed the app on data-enabled tablets and had these tablets available for study participants who did not have a compatible smartphone.

CORA would ideally upload weekly symptom reports directly into patient charts in the electronic health record (EHR) so that oncologists can easily consult them when reviewing a chart before or during the patient’s visit. Unfortunately, logistical limitations of the EHR system in use at the study sites prevented us from delivering symptom reports to clinicians in a way that would be most convenient for them. We sent symptom reports to oncologists by HIPAA-compliant email instead. Thus, we cannot guarantee that oncologists open the symptom report emails that we send them. As more flexible EHRs are developed [29,30] and patient-reported outcomes are integrated into cancer care [31,32] we expect that this barrier to effectively communicating patient symptom trends to oncologists will be removed in future studies.

A methodological limitation is that we did not collect quantitative usage or acceptability data during the development and preliminary testing phase. Surveys probing user acceptability of the CORA app and its modules would have provided useful information for refining the app and justifying its readiness for deployment in an RCT. We have addressed this lack of quantitative feedback data by including app usability and acceptability questions in the RCT. These data will inform how we change and improve future versions of CORA. Likewise, it would have been optimal to collect demographic data from our stakeholders; unfortunately, this was not possible as stakeholders were considered as study team members rather than participants. Ethically, we could not collect personal data from individuals who were not formally consented through IRB processes. However, we did collect demographic information from patients and clinicians who consented as study participants to review the app wireframes. Future studies should seek to create arrangements with funding agencies and IRBs that enable collection of basic demographic information from all stakeholders.

A final limitation is that the version of CORA described in this manuscript does not support all available smartphone operating systems or wearable activity tracking device brands. While integrating more third-party software with CORA entails considerable engineering efforts, it is critical to support the majority of these devices used by individuals with cancer. We intend to make CORA compatible with more types of smartphone devices and wearable activity trackers.

### Comparison With Prior Work

Our development process aligns with the best practices used by teams developing similar apps. Darlow and Wen [16] conducted a review of existing mHealth interventions in oncology and defined a framework for successful mHealth intervention development. Key elements of successful intervention development identified in this review include stakeholder involvement in the development process, addressing the unique needs and experiences of patients and caregivers in the target population, and ensuring user satisfaction with the system along with perceived benefits and limitations. The review also highlights several frameworks for mHealth intervention development, one of which we consider within the theoretical framework of the present intervention development.

Darlow and Wen’s review [16] outlines 8 best practices for the development of mHealth interventions; however, it was published as we were in the final stages of our development process. Despite not being part of our initial aims, our development process met these criteria (Table 4). We engaged stakeholders at all points in development, including health professionals who could ensure that the burden introduced by the intervention would be minimal. Through extensive user testing, we confirmed that our app was simple and intuitive and gave patients a sense of control over their care.

**Table 4:** Best practices of the development phase.

Best practice criteria	Criteria fulfillment in the Chemotherapy Assistant (CORA)	Input from stakeholders to meet criteria
Use a framework for developing mobile health interventions.	Partially met criterion: we consulted the model of medication adherence proposed by Murray et al [18] in designing our intervention. However, we did not use a model for our mobile development process.	Stakeholders did not participate in this aspect of the development process.
Conduct thorough user testing using mixed methods.	Met criterion: we conducted extensive qualitative interviewing through focus groups and individual interviews. We also conducted quantitative surveys with our stakeholders to elicit their feedback. In the second phase of the study, we are collecting quantitative acceptability and usability data.	Stakeholders provided feedback in focus groups and individual interviews during the development process.
Anticipate and plan for the time it will take to carry out the development testing phases, as well as the time it will take to make revisions to a system in between phases.	Met criterion: we allotted time and funding in our initial grant proposal for development testing with revision between phases.	Stakeholders did not participate in this aspect of the development process.
Engage stakeholders throughout the entire development process.	Met criterion: patient, clinician, and community stakeholder feedback was solicited during conceptualization, pilot testing, and final testing of the app.	We engaged stakeholders throughout the development process. Key feedback items from each stakeholder group were implemented into app features (see Table 2).
Ensure that system is simple and that use is intuitive.	Met criterion: we tested an early version of our app prototype with patient and family member stakeholders, eliciting their feedback to inform revisions.	Feedback on wireframes from patient and family stakeholders demonstrated how to simplify the app design.
System component should instill a sense of competence and agency over patient’s own care.	Met criterion: our app enables patients to self-monitor symptom trends and provides advice to help them manage their own symptoms when clinically appropriate.	Feedback from patient and family stakeholders confirmed that our design was acceptable. Feedback from clinicians ensured that patients were provided with timely and accurate information in CORA to manage symptoms.
If health professionals will be needed for system implementation, ensure that any burden is not considerable.	Met criterion: clinicians were included as key stakeholders throughout the development of the app to ensure burden was low. Also, at the start of the trial, we presented our study design to each oncology clinic team at our hospital to ensure clinician acceptability.	Feedback from clinician and practice administrator stakeholders directed how we implemented CORA into clinician workflows.
Publish results of development testing.	Met criterion: this manuscript details the results of our development, pilot testing, and ongoing testing efforts.	Stakeholder feedback collected since completion of the development process has guided how best to describe CORA in this manuscript.

To our knowledge, we are the first to describe the development of an app targeted at promoting adherence to oral chemotherapy for patients with cancer. A recent systematic review describes various interventions to improve adherence to oral chemotherapies; however, none of these interventions is based in mobile technology [11]. We have also described the development of features to address symptom monitoring and management as well as patient-provider communication, which have been included in previous interventions [33-38]. A recent review of mHealth intervention development suggests that these features are the most frequently included in apps designed for cancer patients [16]. Our app enables EMAs of adherence to medication and symptoms. This represents an improvement in validity over methodologies that rely on retrospective reports of symptoms and adherence and addresses the longstanding need for technologies that provide more granular and accurate data reflecting health behaviors [39,40], including adherence [41].

### Lessons Learned and Conclusions

There are a number of ways to streamline app development that we learned in this process. First, it is important to optimize communications between the investigative research team and the technical development team. Initial development of the app, iterating on the design, and fixing bugs may take longer than the study team anticipates. Research and technical teams may have different perspectives on which bugs and features should be prioritized. Based on our experiences, we would advise that study teams anticipate up to 50% more time for development and iteration of software beyond their initial estimates.

Careful planning by the study team members can mitigate difficulties in development. If the initial specifications for the app do not include requirements for reporting of all needed study data, later development of those reporting systems requires a revision. These types of data reporting systems may require significant changes to back-end databases, and thus may cause delays. Furthermore, we found that it is preferable to generate continuous reports of study data rather than infrequent data extractions. Continuous data enables the study team to conduct data quality assurance checks, minimizing potential loss of data occurring due to bugs in the app. Investigators should refer to the eHealth Consolidated Standards of Reporting Trials document to identify data points to collect for accurate reporting on their app [42].

A final lesson learned is the importance of engaging stakeholders and soliciting their feedback. Beginning with our initial conception of an app targeted toward helping patients track their medications, our stakeholders suggested numerous additions and improvements to the app that would enhance the experience for patients and their clinicians. As a result of stakeholder involvement, the final app is patient-centered, user-friendly, and minimally burdensome to clinicians. Stakeholder engagement is an emerging trend not only in development of mHealth apps but in health interventions and patient-centered outcomes research more generally [43], and we believe our development process provides a template for how stakeholders can be engaged to make health interventions more relevant for patients, clinicians, and health care systems alike.

Future directions in the development of our app include pursuing strategies for health-behavior modification that have proven successful in other apps. For example, we could include functionality to integrate other adherence-promoting technologies with our systems. Electronic pill bottles that track when they are opened and closed (as a proxy for when doses are taken) are now widely available as commercial products—for example, the Medication Event Monitoring System. Integrating these technologies with our software could enable patients and clinicians to check via their mobile phones or online portals whether scheduled doses have been taken. Such a system could even upload adherence data to a hospitals electronic medical record and send email or text message notifications to patients and clinicians after missed doses. We also could provide more mechanisms for rewarding user engagement in the app, such as by gamifying the app. Gamification is the application of video game elements, such as embarking on quests and earning trophies or badges, in contexts such as health care [44,45]. Using CORA, a patient could earn an in-app badge or even a real-world reward such as a gift card or discount coupon at a local pharmacy for completing a week with no missed doses.

In addition, given the integral role of caregivers in patient care, features could be added that aid the caregiver in caring for their loved one. For example, a version of CORA designed for caregivers could enable caregivers to set reminders for appointments and medication administration, rate patient symptoms, receive feedback for how to help their loved one manage symptoms, and access tips for caregiver self-care. Future versions of our app could incorporate these and other strategies to improve adherence to oral chemotherapy. Results of our ongoing RCT will inform next steps in app development as well as future research questions.

We have developed a novel app to improve oral chemotherapy adherence and detailed the iterative process here. In developing the app, we engaged stakeholders from across the continuum of care and followed a design process of iteration and usability testing with patients. We will build upon this work through validation of our app in an RCT and developing this app toward wide dissemination to individuals receiving oral chemotherapy treatment.

## Abbreviations

CORA: Chemotherapy Assistant
EHR: electronic health record
EMA: ecological momentary assessment
EMI: ecological momentary intervention
HIPAA: Health Insurance Portability and Accountability Act
IRB: institutional review board
LAMP: Linux, Apache, MySQL, Perl/PHP/Python
MGH: Massachusetts General Hospital
mHealth: mobile health
RCT: randomized controlled trial
UAT: user acceptance testing
